# Automatic QRS complex detection using two-level convolutional neural network

**DOI:** 10.1186/s12938-018-0441-4

**Published:** 2018-01-29

**Authors:** Yande Xiang, Zhitao Lin, Jianyi Meng

**Affiliations:** 1 College of Information Science and Electronic Engineering, Zhejiang University, Zheda Road 38, Hangzhou, 310027 China; 2 Institute of VLSI Design, Zhejiang University, Zheda Road 38, Hangzhou, 310027 China; 3 State Key Laboratory of ASIC and System, Fudan University, Zhangheng Road 825, Shanghai, 201203 China

**Keywords:** Electrocardiogram (ECG), QRS complex detection, Convolutional neural network (CNN)

## Abstract

**Background:**

The QRS complex is the most noticeable feature in the electrocardiogram (ECG) signal, therefore, its detection is critical for ECG signal analysis. The existing detection methods largely depend on hand-crafted manual features and parameters, which may introduce significant computational complexity, especially in the transform domains. In addition, fixed features and parameters are not suitable for detecting various kinds of QRS complexes under different circumstances.

**Methods:**

In this study, based on 1-D convolutional neural network (CNN), an accurate method for QRS complex detection is proposed. The CNN consists of object-level and part-level CNNs for extracting different grained ECG morphological features automatically. All the extracted morphological features are used by multi-layer perceptron (MLP) for QRS complex detection. Additionally, a simple ECG signal preprocessing technique which only contains difference operation in temporal domain is adopted.

**Results:**

Based on the MIT-BIH arrhythmia (MIT-BIH-AR) database, the proposed detection method achieves overall sensitivity *Sen* = 99.77%, positive predictivity rate *PPR* = 99.91%, and detection error rate *DER* = 0.32%. In addition, the performance variation is performed according to different signal-to-noise ratio (SNR) values.

**Conclusions:**

An automatic QRS detection method using two-level 1-D CNN and simple signal preprocessing technique is proposed for QRS complex detection. Compared with the state-of-the-art QRS complex detection approaches, experimental results show that the proposed method acquires comparable accuracy.

## Background

Electrocardiogram (ECG) is a graphical representation of the electric activity of the heart and has been commonly used for cardiovascular disease diagnosis. A typical ECG-based heartbeat mainly consists of three waves including P-wave, QRS complex, and T-wave. The QRS complex is the most prominent feature and it can be used to obtain additional useful clinical information from ECG signals, such as RR interval, QT interval, and PR interval, etc. Thus, QRS detection is critical for ECG-based health evaluation.

The methods of QRS complex detection proposed in the past decades mainly consist of the preprocessing stage and the decision-making stage.

The preprocessing stage comprising nonlinear and/or linear filtering aims at reducing noise and facilitating lexical analysis afterwards. The preprocessing approaches in many previous studies mainly adopt linear filtering and wavelet transform for noise removal in ECG [1–5]. After filtering, the signals are further processed through numerous techniques, such as moving average filter [1, 6], squaring function [7], and Hilbert transform [8].

The preprocessing stage is followed by decision stage where the envelope of a signal is extracted and the final QRS complex location is decided. The decision-making stage usually adopts heuristic methods to detect the real QRS complex location. A number of algorithms based on derivative [8, 9], digital filters [10], and wavelet transform [11] have frequently been used for QRS detection. With the improvement of hardware environment, much more methods adopt wavelet transforms. In wavelet-based techniques, the efficiency of wavelet transform strongly depends on the choice of the mother wavelets. Other detection algorithms proposed in the literatures including mathematical morphology [12], hidden Markov model [13], S-transform [14], Hilbert transform [2], regular grammar [15], quadratic filter [16], multiresolution entropy [17], sparse representation [18], and singular value decomposition (SVD) [19]. Although the above detection methods present high accuracy with their experimental datasets, their performance largely depends on selected mother wavelets in wavelet transform as well as knowledge-based and fixed parameters in other methods. Therefore, in case of ECG patterns, which are physiological variations due to times, individuals, or circumstances, the choice of appropriate mother wavelets and parameters becomes difficult. In addition, extracting hand-crafted features manually for QRS complex detection may introduce significant computational complexity of overall process, especially in the transform domains.

To adapt with various morphologies of ECG signals, some algorithms adopt adaptive threshold which is a very important parameter in QRS complex detection. There are two categories of adaptive threshold, including single level [20–23] and multiple levels [24, 25]. However the adaptive threshold helps to improve detection accuracy at the expense of computational complexity, which makes it difficult for real-time QRS detection. Artificial neural network (ANN) based approaches have been proposed for real-time detection [26, 27]. Based on ECG signals in the CSE Data Set-3 library [28], Vijaya et al. [26] employs ANN for R-wave detection. Only 1491 QRS complexes are used for performance evaluation, which makes it difficult to prove the robustness of the method. Arbateni et al. [27] utilizes ANN-based whitening filter, matched filter, squaring and moving average filter for ECG preprocessing. Then the position of QRS complex is located by decision logic. Although the algorithm achieves an average detection error rate of 0.28%, it introduces much computational complexity for processing ECG signals. This may hinder the detection method from the usage in the light-weight healthcare devices.

In order to solve the drawbacks mentioned above, an attention-based two-level 1-D convolutional neural network (CNN) is proposed for extracting morphological features of QRS complex automatically. CNNs have achieved the state-of-the-art performance in deep learning tasks [29, 30]. It is also worth noting that visual attention models have been applied in computer vision problems for fine-grained object detection [31] and fine-grained categorization [32]. The attention model is able to process candidate regions for classification with different resolution and reduce processing cost by focusing on a restricted set of regions. With the help of the attention model, discriminatory power could be focused on the specific parts of the input pattern, which helps to classify the pattern accurately [33].

For reducing computational cost, adapting with variations of ECG signals, discarding hand-crafted features, and improving accuracy of QRS detection, an accurate method for QRS detection based on CNN is proposed. In our context, two-level CNN comprised of object-level and part-level CNNs is adopted to extract ECG morphological features for QRS complex detection. To our knowledge, this is the first study where the two-level 1-D CNN is used for ECG-based QRS complex detection. In addition, only difference and averaging operations are applied for ECG signal preprocessing.

## Materials and overview

### ECG samples

The ECG signals in the MIT-BIH arrhythmia (MIT-BIH-AR) database and the St. Petersburg Institute of Cardiological Technics 12-lead Arrhythmia (INCART) database are used in this study. We divide the signals of the MIT-BIH-AR database into training part and testing part, and adopt the signals of the INCART database to estimate the robustness of the proposed detection method.

The MIT-BIH-AR database contains 48 ECG recordings from 47 subjects, and each recording is sampled at 360 Hz for 30 min with 11-bit resolution over a 10 mV range. Each recording comprises two ECG leads, one lead is modified-lead II (MLII) and the other lead is mainly lead V1, sometimes V2, V4 or V5, which are summarized in Table 1. In this study, only MLII is used, and therefore record 102 and record 104 are not taken into consideration. The database consists of annotations for both heartbeat class information and R-wave position information verified by two or more expert cardiologists. All beats of the database are assigned corresponding labels by using a 17-label set. The INCART database contains 75 annotated recordings of 12-lead ECG signals. Each of them is 30 min long, sampled at 257 Hz, and gained varying from 250 to 1100 analog-to-digital converter (ADC) units per 1 mV. To match with the MIT-BIH-AR database, each recording of the INCART database should be resampled at 360 Hz. This database contains over 175,000 annotated beats.

**Table 1:** Summary of the channel distribution of the records in the MIT-BIH-AR database

Channel 1	Channel 2	Record
MLII	V1	101 105 106 107 108 109 111 112
		113 115 116 118 119 121 122 200
		201 202 203 205 207 208 209 210
		212 213 214 215 217 219 220 221
		222 223 228 230 231 232 233 234
MLII	V2	103 117
MLII	V4	124
MLII	V5	100 123
V5	MLII	114
V5	V2	102 104

### Overview of the proposed method

**Fig. 1:**
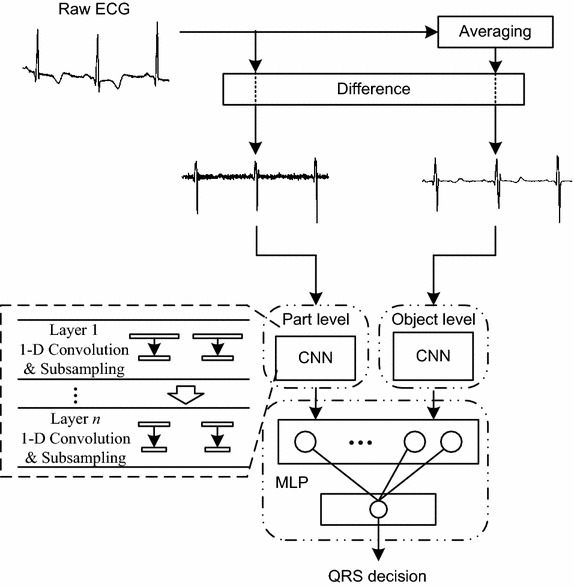
Overview of the proposed QRS complex detection method

The proposed method consists of three steps, including signal preprocessing, feature extraction, and QRS complex location decision. The overview diagram is shown in Fig. 1. First, difference operation and averaging operation are used to process raw ECG signals. The outputs of this step are sent to two-level 1-D CNN for feature extraction. The two-level CNN is applied to focus on different parts of the ECG signals and extract different grained morphological features. The CNN adopts hierarchical architecture, and each layer consists of 1-D convolution and 1-D subsampling. The coarse-grained features are extracted by object-level CNN and the fine-grained features are extracted by part-level CNN. All the extracted features are fed into MLP for QRS complex location decision.

## Methods

### Preprocessing

**Fig. 2:**
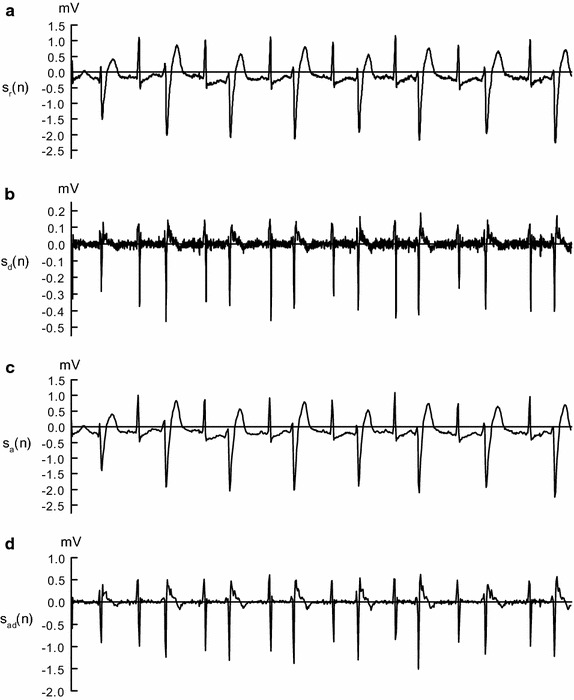
Outputs obtained at preprocessing stage of the proposed method. **a** Raw ECG signal from record 200 in the MIT-BIH-AR database; **b** signal obtained by difference operation; **c** signal obtained by averaging operation; **d** signal obtained by averaging operation followed by difference operation

The raw ECG signal $s_r(n)$ is differentiated to accentuate the QRS complex which is characterized by a high slope. The difference ECG signal is obtained by making subtraction between adjacent samples, which is shown as follows:

1 $$\begin{aligned} s_d[n]=s_r[n]-s_r[n-1] \end{aligned}$$

where $s_r[n]$ is the raw ECG signal data at time *n*, and $s_d[n]$ is the difference data at time *n*. The two signals are illustrated in Fig. 2a, b. It is not necessary to normalize ECG signal as other methods do [34–36]. Then, the difference signal are sent to part-level CNN for fine-grained feature extraction.

In addition, the raw ECG signal $s_r(n)$ is averaged per several adjacent samples, which is followed by difference operation. Then, the difference signal is fed into object-level CNN for coarse-grained feature extraction. The averaging and difference operations are represented as follows:

2 $$\begin{aligned} s_a[n]=\frac{1}{N_i}\sum _{i=1}^{N_i}{s_r[n*N_i+i]} \end{aligned}$$

3 $$\begin{aligned} s_{ad}[n]=s_a[n]-s_a[n-1] \end{aligned}$$

where $s_a(n)$ represents the average ECG signal shown in Fig. 2c, and $s_{ad}(n)$ represents the average difference signal shown in Fig. 2d. The number of samples in $s_r(n)$ is $N_i$ times more than that in $s_a(n)$.

### ECG signal segmentation

**Fig. 3:**
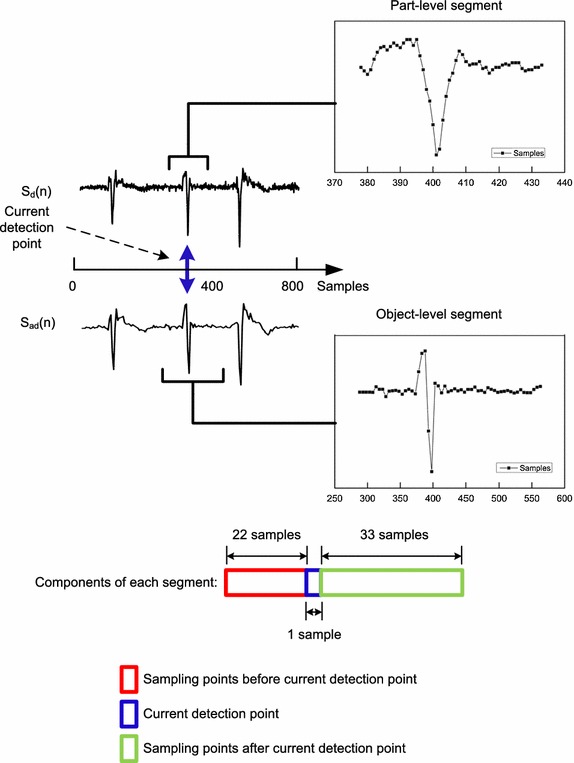
Segment method used for ECG signal

A heartbeat is commonly composed of P-wave, QRS complex, and T-wave. Therefore, we choose segment length of 56 sampling points from difference and average difference ECG signal respectively, which is shown in Fig. 3. 22 sampling points before the current detection point and 33 sampling points after it. Given the raw ECG signal in the MIT-BIH-AR database sampled at 360 Hz, all of the 56 samples equivalent to 0.78 s in average difference signal and 0.16 s in difference signal. In this way, it can cover a whole heartbeat cycle as well as QRS complex when the current detection point is R-peak.

### Attention-based two-level feature extraction and QRS detection

**Fig. 4:**
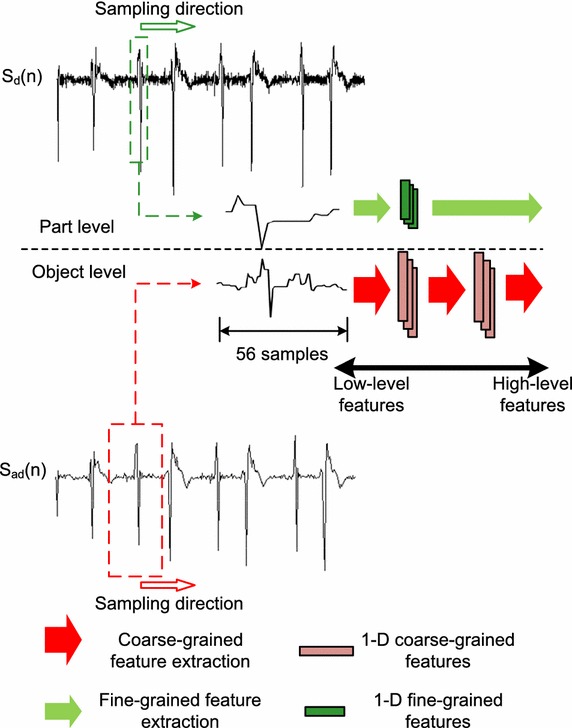
Structure of attention-based two-level 1-D CNN

To extract different grained morphological features from difference ECG signal as well as average difference ECG signal, an attention-based automatic feature extraction system comprised of object-level 1-D CNN and part-level 1-D CNN is proposed, which is shown in Fig. 4. The object-level CNN is applied to extract coarse-grained features corresponding to object-level segment. The segmented ECG signal is preprocessed by averaging and difference operations. The part-level CNN is used to extract fine-grained features by focusing attention on part-level segment. The ECG signal of this segment is preprocessed by difference operation only. The two levels of the system are combined for training with back-propagation (BP) scheme. The two-level CNN adopts hierarchical structure, in which different abstract features are extracted from different layers. In the low-level layer, low-level features are extracted. Then, the extracted features are propagated to the next hidden layer for extracting higher-level features. Each layer of the two-level CNN consists of convolution stage and subsampling stage. Convolution stage applies convolution operation to the input, and then output the result to the next stage. Weights are shared and several feature maps can be computed at the stage. Subsampling stage combines the outputs of clustered neurons at convolution stage into one. There are mainly two kinds of subsampling operations including max-subsampling and mean-subsampling. Max-subsampling outputs the maximum value of clustered neurons. Mean-subsampling outputs the average value of clustered neurons. In this study, mean-subsampling operation is adopted. In order to extract different grained features corresponding to different-level segmentation, the number of layers between part-level and object-level CNNs can be implementation defined. All of the features extracted by the two-level CNN are concatenated and sent to MLP for final QRS complex detection.

**Fig. 5:**
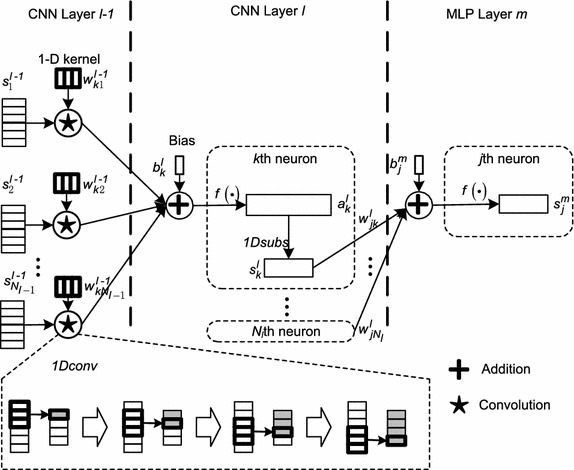
1-D CNN structure in the proposed detection system

The QRS detection process is composed of two steps including training and decision. The training process is utilized for optimizing weights and biases, and then the neural network configured with the trained weights and biases are used to detect QRS complexes. The relationships among CNN and MLP layers are presented in Fig. 5.

The intermediate value as well as final output of the *k*th neuron at CNN layer *l* are computed as Eqs. 4 and 5 respectively, and the output of the *j*th neuron at MLP layer *m* is computed as Eq. 6.

4 $$\begin{aligned} a_k^l=f\left(\sum _{i=1}^{N_{l-1}}1Dconv(w_{ki}^{l-1},\,s_i^{l-1})+b_k^l\right) \end{aligned}$$

5 $$\begin{aligned} s_k^l=1Dsubs(a_k^l) \end{aligned}$$

6 $$\begin{aligned} s_j^m=f\left(\sum _{k=1}^{N_l}w_{jk}^{l}s_k^l+b_j^m\right) \end{aligned}$$

where $w_{ki}^{l-1}$ is 1-D weight kernel between *k*th neuron at layer *l* and *i*th neuron at layer $l-1.$ $s_i^{l-1}$ is the output of *i*th neuron at layer $l-1.$ $a_k^l$ is the intermediate activation value of *k*th neuron at layer *l*. $b_k^l$ is the bias value of *k*th neuron at layer *l*. 1*Dconv* and 1*Dsubs* represent 1-D convolution and 1-D subsampling respectively. *f*(.) represents activation function. In this study, the rectified linear unit (Relu) activation function is adopted in CNN layers as well as MLP hidden layers, and Softmax activation function is used in MLP output layer for final QRS complex location decision. The goal of training is to minimize objective function *E* by adjusting the kernel weights and biases:

7 $$\begin{aligned} E=E(y_1,\,y_2,...,y_N)=\sum _{j=1}^N\left(t_j-y_j\right)^2 \end{aligned}$$

where *N* is the total neuron number of output layer. For a given input vector $\varvec{v},$ $[t_1,\,t_2,\ldots ,t_N]$ and $[y_1,\,y_2,\ldots ,y_N]$ are the corresponding target output vector and predicted output vector respectively. The weights and the biases are updated with the learning rate $\eta$ as represented in Eqs. 8 and 9. In this study, we set the initial learning rate as $\eta =0.005,$ and slightly decrease it by 0.0001% during each learning iteration.

8 $$\begin{aligned} w_{ki}^l(t)=w_{ki}^l(t-1)-\eta \frac{\partial E}{\partial w_{ki}^l(t-1)} \end{aligned}$$

9 $$\begin{aligned} b_k^l(t)=b_k^l(t-1)-\eta \frac{\partial E}{\partial b_k^l(t-1)} \end{aligned}$$

After the two-level CNN as well as the MLP are trained, both a given difference ECG signal and its corresponding average difference ECG signal are sent to the neural network system for QRS position decision.

## Results

As mentioned above, for the two-level CNN, it is feasible to adopt different depths for focusing on different-level segmentations. Two CNN layers are used for object-level feature extraction, and one CNN layer is used for part-level feature extraction. The detail configuration of the two-level CNN is described in Table 2. The outputs of the two levels are concentrated and sent into two-layer MLP for QRS location decision. The first MLP layer contains 20 neurons which are fully connected with neuron of the following layer. In this study, we find that the accuracy of QRS detection is not improved while the number of neurons in the first MLP layer exceeds 20. The second MLP layer contains four neuron used for QRS detection. The four neurons are adopted to detect Q wave, R wave, S wave and non-QRS segment respectively.

**Table 2:** Detail description of the proposed attention-based two-level 1-D CNN configuration

		Object-level CNN	Part-level CNN
CNN layer 1			
1-D convolution kernel length		5	5
1-D subsampling factor		2	2
Number of neurons		5	5
CNN layer 2			
1-D convolution kernel length		5	None
1-D subsampling factor		2	None
Number of neurons		5	None

The preprocessing and segmentation are processed by Matlab, and the neural network is trained by using high-level Python library Keras [37]. Keras allows for easy and fast prototyping of the neural networks.

For training the proposed two-level CNN, 400 representative QRS complexes, along with their associated non-QRS segments are selected from the MIT-BIH-AR database. The ECG signals for testing contain 46 ECG records from the MIT-BIH-AR database as well as all ECG records from the INCART database. Only MLII of the MIT-BIH-AR database and lead II of the INCART database are used. The measured metrics adopted for evaluating detection performance are sensitivity (Sen), positive predictivity rate (PPR), detection error rate (DER), and accuracy (Acc), which are calculated by:

10 $$\begin{aligned} Sen\,(\%) = \frac{TP}{TP+FN}*100 \end{aligned}$$

11 $$\begin{aligned} PPR\,(\%) = \frac{TP}{TP+FP}*100 \end{aligned}$$

12 $$\begin{aligned} DER\,(\%) = \frac{FN+FP}{TP+FN}*100 \end{aligned}$$

13 $$\begin{aligned} Acc\,(\%) = \frac{TP}{TP+FP+FN}*100 \end{aligned}$$

The above four metrics are computed by the quantity of true positive (TP), false positive (FP), and false negative (FN). TP is number of correct QRS prediction. FP is number of incorrect QRS prediction. FN is number of incorrectly rejected QRS.

The QRS complex detection performance achieved by using the 46 ECG records in the MIT-BIH-AR database is shown in Table 3. As the table shows, the overall *Sen* = 99.77%, *PPR* = 99.91%, and *DER* = 0.32%. All the *Sen* and *PPR* values are higher than 99%. For 20 cases of detection in the MIT-BIH-AR database, *Sen* values are 100.00%. For 23 cases of detection in the database, *PPR* values are 100.00%. In addition, only 4 cases have error values greater than 1%. These records are 106, 223, 228, and 233. The number of undetected and false positive QRS complexes results in the consequence. Records where the error values exceed 1% are mainly due to two reasons: the morphology of Q-wave is much like the R-wave in some segmentations or in other cases the slope of QRS complex is quite gentle.

**Table 3:** Performance evaluation of the proposed method using the MIT-BIH-AR database

Record	Total beats	TP	FP	FN	Sen (%)	PPR (%)	DER (%)	Acc (%)
100	2273	2273	0	0	100.00	100.00	0.00	100.00
101	1865	1865	0	0	100.00	100.00	0.00	100.00
103	2084	2084	0	0	100.00	100.00	0.00	100.00
105	2572	2560	0	12	99.53	100.00	0.47	99.53
106	2027	2010	8	17	99.16	99.60	1.23	98.77
107	2137	2137	0	0	100.00	100.00	0.00	100.00
108	1763	1763	0	0	100.00	100.00	0.00	100.00
109	2532	2523	7	9	99.64	99.72	0.63	99.37
111	2124	2124	0	0	100.00	100.00	0.00	100.00
112	2539	2539	0	0	100.00	100.00	0.00	100.00
113	1795	1795	0	0	100.00	100.00	0.00	100.00
114	1879	1872	5	7	99.63	99.73	0.64	99.36
115	1953	1953	0	0	100.00	100.00	0.00	100.00
116	2412	2398	6	14	99.42	99.75	0.83	99.17
117	1535	1535	0	0	100.00	100.00	0.00	100.00
118	2278	2277	1	1	99.96	99.96	0.09	99.91
119	1987	1976	8	11	99.45	99.60	0.96	99.05
121	1863	1863	0	0	100.00	100.00	0.00	100.00
122	2476	2476	0	0	100.00	100.00	0.00	100.00
123	1518	1517	2	1	99.93	99.87	0.20	99.80
124	1619	1617	5	2	99.88	99.69	0.43	99.57
200	2601	2596	1	5	99.81	99.96	0.23	99.77
201	1963	1962	0	1	99.95	100.00	0.05	99.95
202	2136	2136	0	0	100.00	100.00	0.00	100.00
203	2980	2960	5	20	99.33	99.83	0.84	99.16
205	2656	2656	0	0	100.00	100.00	0.00	100.00
207	1860	1856	0	4	99.78	100.00	0.22	99.78
208	2955	2948	2	7	99.76	99.93	0.30	99.70
209	3005	3002	2	3	99.90	99.93	0.17	99.83
210	2650	2634	3	16	99.40	99.89	0.72	99.28
212	2748	2748	0	0	100.00	100.00	0.00	100.00
213	3251	3242	2	9	99.72	99.94	0.34	99.66
214	2262	2256	1	6	99.73	99.96	0.31	99.69
215	3363	3357	3	6	99.82	99.91	0.27	99.73
217	2208	2200	5	8	99.64	99.77	0.59	99.41
219	2154	2150	1	4	99.81	99.95	0.23	99.77
220	2048	2048	0	0	100.00	100.00	0.00	100.00
221	2427	2413	9	14	99.42	99.63	0.95	99.06
222	2483	2483	0	0	100.00	100.00	0.00	100.00
223	2605	2587	9	18	99.31	99.65	1.04	98.97
228	2053	2034	6	19	99.07	99.71	1.22	98.79
230	2256	2256	0	0	100.00	100.00	0.00	100.00
231	1571	1571	0	0	100.00	100.00	0.00	100.00
232	1780	1780	1	0	100.00	99.94	0.06	99.94
233	3079	3053	7	26	99.16	99.77	1.07	98.93
234	2753	2752	0	1	99.96	100.00	0.04	99.96
Overall	105,078	104,837	99	241	99.77	99.91	0.32	99.68

The performance comparison between the proposed method and other eight state-of-the-art approaches is shown in Table 4. The *DER* value reflects the general performance of these approaches, and thus the order of each algorithm is sorted based on this index. As presented in the table, for the MIT-BIH-AR database, the performance of our proposed method is comparable to other state-of-the-art algorithms. The maximal and minimal average *DER* values are 0.51 and 0.25% respectively.

**Table 4:** Comparison of performance of several QRS detection methods using the MIT-BIH-AR database

Method	TP	FP	FN	Sen (%)	PPR (%)	DER (%)
Zidelmal et al. [14]	108,323	97	171	99.84	99.91	0.25
Arbateni and Bennia [27]	109,273	109	210	99.82	99.91	0.29
Kholkhal and Reguig [23]	106,310	48	259	99.76	99.95	0.29
This work	104,837	99	241	99.77	99.91	0.32
Zhou et al. [18]	109,224	139	213	99.81	99.87	0.32
Bouaziz et al. [4]	109,354	232	140	99.87	99.79	0.34
Phukpattaranont [16]	109,281	210	202	99.82	99.81	0.38
Farashi [17]	109,692	163	273	99.75	99.85	0.39
Hamdi et al. [15]	73,278	92	284	99.74	99.86	0.51

**Fig. 6:**
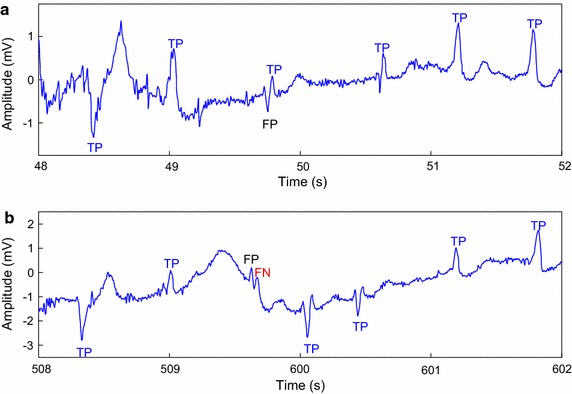
Examples of incorrect detection in record 203

The representative examples of incorrect detection performed by the proposed detection method are shown in Fig. 6. The record 203 in the MIT-BIH-AR database contains much noise and irregular heartbeats in the aspect of morphology, which leads to a considerable number of FNs and FPs.

**Fig. 7:**
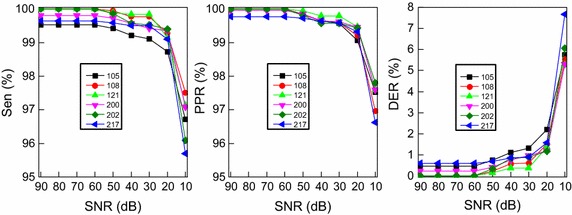
Sen variation according to different SNR values based on 105, 108, 121, 200, 202, and 217 ECG records

Table 5 shows comparison result of the *DER* values by evaluating records 105, 108, 121, 200, 202, and 217 from the MIT-BIH-AR database. The best result for each record is emphasized by italicface. The *DER* values of records 108, 121, and 202 from the proposed algorithm are minimal at 0.00%. In addition, to assess the robustness of the proposed detection method to noise, we add Gaussian noise with 9 different signal-to-noise ratio (SNR) values to the raw ECG signals including records 105, 108, 121, 200, 202, and 217, and the corresponding detection results are presented in Table 6. As to SNR value greater than 30 dB, the proposed detection method provides high *Sen* values which exceed 99%. As to SNR value greater than 20 dB, the method provides high *PPR* values which exceed 99%. Fig. 7 presents the variation of *Sen*, *PPR*, and *DER* values depending on different SNR values. Compared with detection performance using noise-free ECG signals, both the *Sen* and *PPR* values are close to the values without noise when the SNR value is larger than 10 dB. The reasons behind the robustness to noise mainly focus on two reasons. One is that the proposed signal preprocessing approach can eliminate noise to some extent. The other is that the ECG signals used at the training stage are also combined with noise, so that the proposed method can extract features of ECG signals and detect QRS complexes with noise.

**Table 5:** Comparison of DER values on 105, 108, 121, 200, 202, and 217 records of the MIT-BIH-AR database

Method	105	108	121	200	202	217
This work	0.47	*0.00*	*0.00*	0.23	*0.00*	0.59
Zidelmal et al. [14]	1.24	2.44	0.16	0.23	0.09	0.23
Arbateni and Bennia [27]	0.23	0.51	0.16	0.31	0.33	0.64
Kholkhal and Reguig [23]	0.37	0.53	0.11	0.37	0.05	0.14
Zhou et al. [18]	1.48	2.72	0.05	0.66	0.28	*0.09*
Bouaziz et al. [4]	0.81	8.40	0.10	0.30	0.09	0.23
Phukpattaranont [16]	1.59	4.08	*0.00*	0.19	*0.00*	0.27
Farashi [17]	2.60	2.95	0.16	0.81	1.17	0.27
Hamdi et al. [15]	*0.04*	0.11	0.27	*0.00*	1.17	*0.09*

Best results are italicized

**Table 6:** Detection performance with noise added and without noise

Record	Index	Without noise	SNR in noise added (dB)								
			90	80	70	60	50	40	30	20	10
105	Sen (%)	99.53	99.53	99.53	99.53	99.53	99.42	99.22	99.11	98.73	96.71
	PPR (%)	100.00	100.00	100.00	100.00	100.00	99.84	99.65	99.57	99.07	97.52
	DER (%)	0.47	0.47	0.47	0.47	0.47	0.74	1.12	1.32	2.20	5.74
108	Sen (%)	100.00	100.00	100.00	100.00	100.00	99.94	99.77	99.77	99.27	97.51
	PPR (%)	100.00	100.00	100.00	100.00	100.00	99.83	99.66	99.60	99.21	96.97
	DER (%)	0.00	0.00	0.00	0.00	0.00	0.23	0.57	0.62	1.52	5.53
121	Sen (%)	100.00	100.00	100.00	100.00	100.00	99.89	99.84	99.84	99.15	97.13
	PPR (%)	100.00	100.00	100.00	100.00	100.00	99.95	99.79	99.79	99.47	97.59
	DER (%)	0.00	0.00	0.00	0.00	0.00	0.16	0.38	0.38	1.38	5.27
200	Sen (%)	99.81	99.81	99.81	99.81	99.81	99.73	99.58	99.43	99.35	97.08
	PPR (%)	99.96	99.96	99.96	99.96	99.96	99.85	99.65	99.62	99.46	97.59
	DER (%)	0.23	0.23	0.23	0.23	0.23	0.42	0.77	0.96	1.19	5.31
202	Sen (%)	100.00	100.00	100.00	100.00	100.00	99.86	99.58	99.53	99.40	96.09
	PPR (%)	100.00	100.00	100.00	100.00	100.00	99.81	99.58	99.58	99.44	97.80
	DER (%)	0.00	0.00	0.00	0.00	0.00	0.33	0.84	0.89	1.16	6.07
217	Sen (%)	99.64	99.64	99.64	99.64	99.64	99.59	99.50	99.50	99.10	95.69
	PPR (%)	99.77	99.77	99.77	99.77	99.77	99.73	99.64	99.59	99.32	96.62
	DER (%)	0.59	0.59	0.59	0.59	0.59	0.68	0.86	0.90	1.58	7.66

In this study, more than 170,000 beats of the INCART database are also used to estimate the capability of the proposed QRS detection method. As presented in Table 7, based on the database, the proposed technique achieves *Sen* = 99.86%, *PPR *= 99.89%, *DER *= 0.25%, and *Acc* = 99.75%.

**Table 7:** Performance evaluation of the proposed method using the INCART database

Total beats	TP	FP	FN	Sen (%)	PPR (%)	DER (%)	Acc (%)
175,914	175,660	189	254	99.86	99.89	0.25	99.75

The proposed detection method is also evaluated in the aspect of computational complexity. A computer with Intel Core i3 CPU 3.5 GHz is used for evaluation. For example, the time consumed for the QRS detection on 30-min ECG record 100 (2273 beats) of the MIT-BIH-AR database is 14.53 s, which is faster than other state-of-the-art approaches [5, 23]. The method presented by Karimipour [5] takes more than 8 ms to detect one QRS complex and Mourad [23] takes 141 s for QRS detection for the same record. The outperformance is due to the proposed simple signal preprocessing technique and light-weight CNN.

## Discussion

QRS complex is the most protruding feature in the ECG with R-peak as the most significant wave. With the help of QRS detection, other components in the ECG signals can be found, such as P wave, T wave, RR interval and PR interval, etc. [11]. Also, QRS detection can provide useful information for biological signal processing, such as heartbeat classification [38], the heart rate computation [39], ECG compression [40], biometrics [41], etc. Although QRS detection is important, the diagnoses of some abnormalities do not have to detect QRS. In the study of Acharya et al. [34, 35], CNN is used for automatic arrhythmia and coronary artery disease detection. All the raw ECG signals are downsampled and removed noise at first. Then, the ECG signals are separated into two different durations of segments and sent to corresponding CNNs for final arrhythmia classification.

The detection for QRS complex as well as many abnormalities in the ECG is complicated at the presence of noise. However, the noise can be used to improve the detection robustness to noise. One of the reasons behind the robustness to noise of the proposed method is that the ECG signals used at the training stage are combined with noise. Similarly, in the study of Acharya et al. [36], both the noisy and the denoised ECG signals are segmented using the detected R-peaks and then sent to CNN for training. Although the noise reduces the overall performance, the trained CNN can be implemented for detection of abnormalities with and without noise.

In order to facilitate feature extraction and reduce computational complexity, only difference and averaging operations are applied for preprocessing in the proposed method. Then, the two-level 1-D CNN is used for automatic extracting different grained morphological features which are sent to MLP for final QRS detection. The performance of our proposed method is comparable to the performances showed in Tables 4 and 5. The robustness to noise of the proposed method is also assessed and shown in Table 6.

The advantages of our proposed method are summarized below:

1. Both the feature extraction and final QRS detection are automatic by using two-level CNN and MLP.
2. The computation cost of the proposed QRS detection method is low.
3. The proposed detection method is robust to noise.

The shortages of our proposed method are as follows:

1. The training of the proposed method is a time-consuming process.
2. The length of input ECG signal is fixed once the structures of the CNN and the MLP are determined.

## Conclusion

In this paper, an automatic QRS complex detection method is proposed, which adopts morphological features of ECG signals. The coarse-grained and fine-grained morphological features are extracted using attention-based two-level 1-D CNN, negating the necessity to extract features manually. In order to facilitate feature extraction mentioned above, a simple preprocessing technique is also adopted. The experimental results obtained by using the MIT-BIH-AR database and the INCART database show that the proposed QRS detection method provides robustness to noise and high detection performance.

Based on the identified QRS complex, the rest of the ECG waves can be detected. The work assists medical diagnoses and serves as an entry point to almost all automated ECG analysis. In future work, we will combine ECG signals and other biomedical signals to improve the accuracy and the robustness to noise of QRS complex detection. In addition, the detected waves will be used for heartbeat classification which is crucial in cardiovascular disease diagnosis.

## Abbreviations

1-D: one-dimensional
Acc: accuracy
ADC: analog-to-digital converter
ANN: artificial neural network
BP: back-propagation
CNN: convolutional neural network
DER: detection error rate
ECG: electrocardiogram
INCART: St. Petersburg Institute of Cardiological Technics 12-lead Arrhythmia
MIT-BIH-AR: MIT-BIH arrhythmia
MLII: modified-lead II
MLP: multi-layer perceptron
PPR: positive predictivity rate
Relu: rectified linear unit
Sen: sensitivity
SNR: signal-to-noise ratio
SVD: singular value decomposition
TP: true positive
FP: false positive
FN: false negative
